# LMO7 as an Unrecognized Factor Promoting Pancreatic Cancer Progression and Metastasis

**DOI:** 10.3389/fcell.2021.647387

**Published:** 2021-03-08

**Authors:** Xinjian Liu, Hao Yuan, Jing Zhou, Qiongling Wang, Xiaoqiang Qi, Catharine Bernal, Diego Avella, Jussuf T. Kaifi, Eric T. Kimchi, Parrett Timothy, Kun Cheng, Yi Miao, Kuirong Jiang, Guangfu Li

**Affiliations:** ^1^Department of Surgery, University of Missouri-Columbia, Columbia, MO, United States; ^2^Department of Pathogen Biology, Key Laboratory of Antibody Technique of National Health Commission of China, Nanjing Medical University, Nanjing, China; ^3^Pancreas Center, The First Affiliated Hospital of Nanjing Medical University, Nanjing, China; ^4^Ellis Fischel Cancer Center, University of Missouri-Columbia, Columbia, MO, United States; ^5^Department of Pathology and Anatomical Sciences, University of Missouri-Columbia, Columbia, MO, United States; ^6^Division of Pharmacology and Pharmaceutical Sciences, School of Pharmacy, University of Missouri-Kansas City, Kansas City, MO, United States; ^7^Department of Molecular Microbiology and Immunology, University of Missouri, Columbia, MO, United States

**Keywords:** pancreatic cancer, LIM domain only 7 (LMO7), cell cycle, apoptosis, CRISPR-Cas9

## Abstract

Pancreatic cancer (PC) is one of the most lethal human malignancies without effective treatment. In an effort to discover key genes and molecular pathways underlying PC growth, we have identified LIM domain only 7 (LMO7) as an under-investigated molecule, which highly expresses in primary and metastatic human and mouse PC with the potential of impacting PC tumorigenesis and metastasis. Using genetic methods with siRNA, shRNA, and CRISPR-Cas9, we have successfully generated stable mouse PC cells with LMO7 knockdown or knockout. Using these cells with loss of LMO7 function, we have demonstrated that intrinsic LMO7 defect significantly suppresses PC cell proliferation, anchorage-free colony formation, and mobility *in vitro* and slows orthotopic PC tumor growth and metastasis *in vivo*. Mechanistic studies demonstrated that loss of LMO7 function causes PC cell-cycle arrest and apoptosis. These data indicate that LMO7 functions as an independent and unrecognized druggable factor significantly impacting PC growth and metastasis, which could be harnessed for developing a new targeted therapy for PC.

## Introduction

Pancreatic cancer (PC) is one of the most lethal malignancies with an average 5-year survival rate of <6% (Ferlay et al., 2010; Siegel et al., 2012; Chu et al., 2018). The American Cancer Society estimates that 57,600 people will be diagnosed with PC and 47,050 of them will die in the US in 2020 (Ren et al., 2018; Siegel et al., 2020). Surgery is a curable treatment but only eligible for 10%–20% of patients. Majority of patients are diagnosed at the late stage with tumor metastasis at which point surgical treatment is minimally effective (Kim et al., 2018). Therefore, developing a novel therapeutic strategy for PC treatment is urgently required.

Over the past 15 years, the therapeutic paradigm in cancer treatment has shifted from traditional cytotoxic chemotherapy to targeted therapy. Many of them have been approved by the Food and Drug Administration (FDA) to treat different cancers (Sawyers, 2004). Imatinib is one outstanding example which is used in the treatment of chronic myelogenous leukemia (CML) with a significant therapeutic effect by specifically inhibiting tyrosine kinases BCR-ABL, c-KIT, and PDGFRA (Iurlo et al., 2015). Previous studies have identified some critical signaling pathways mediating PC initiation and progression which significantly improve our understanding of PC (Tanaka, 2016). Targeting key factors involved in EGFR, RAS/RAF/MEK, PI3K/AKT, WNT, and HEDGEHOG signaling pathways has resulted in the generation of various targeted therapies (Thayer et al., 2003; Wong and Lemoine, 2009). However, the ongoing and completed targeted therapies focusing on these pathways failed to demonstrate efficacy in PC (di Marco et al., 2016) with a median overall survival of less than one year (Amanam and Chung, 2018). Thus, we propose to identify under-investigated molecules important for PC initiation, progression, and metastasis in order to develop new powerful molecularly targeted therapy (MTT) against PC (O’Brien, 1996).

In an effort to discover key genes important for PC progression or suppression, we have identified some under-investigated molecules significantly impacting PC tumorigenesis and metastasis (Qian et al., 2017; Liu et al., 2019). Here, we report another novel finding about LIM domain only 7 (LMO7). LMO7 is a member of PDZ and the LIM domain-containing protein family, implicated in cancer-related pathological conditions and the regulation of cell adhesion (Ooshio et al., 2004; Hu et al., 2011). While having only low or limited expression in a few tissues, LMO7 has been found to upregulate in colorectal, breast, lung, thyroid, and liver cancer and plays a critical role in cancer metastasis (Nakamura et al., 2005, 2011; Hu et al., 2011; He H. et al., 2018). Recent studies demonstrated that LMO7 may be involved in recurrent chromosomal rearrangements of FRK in inflammatory hepatocellular adenomas (Bayard et al., 2020) and serve as a biomarker in common chronic lung diseases (Maghsoudloo et al., 2020). Last year, another group discovered that LMO7 is specifically localized in the cuticular plate and the cell junction. LMO7 knockout mice suffer multiple cuticular plate deficiencies, including reduced filamentous actin density and abnormal stereocilia rootlets (Du et al., 2019). The elevated levels of LMO7, also known as PCD1, have been reported in PC (Kang et al., 2000). However, no report has been found to describe the role of LMO7 in PC carcinogenesis, progression, and metastasis. In addition, the current studies about the role of LMO7 on cancers generated controversial information (Kainu et al., 2000; Rozenblum et al., 2002). We proposed to investigate the contribution of LMO7 on PC and elucidate the underlying mechanisms.

In this study, we have applied different strategies to generate stable PC cells with LMO7 knockdown or knockout. Using these cells and their formed orthotopic tumors, we evaluated how intrinsic LMO7 defect impacts PC tumor progression and metastasis as well as the underlying mechanisms. Our findings suggest that LMO7 plays an important role in PC and represents a novel and druggable molecule target for PC.

## Results

### PC Progression in Human and Mice Is Associated With the Increased Expression of LMO7

**FIGURE 1:**
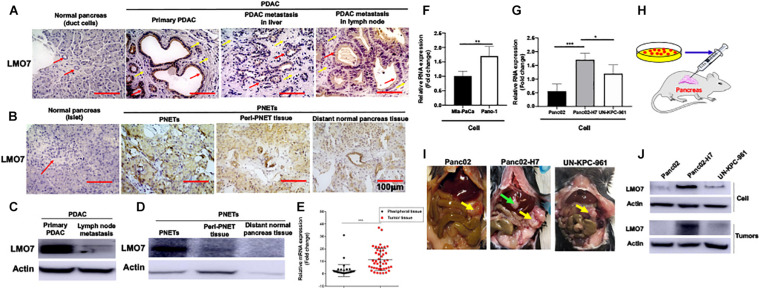
Increased expression of LMO7 protein and mRNA in human and mouse PC tumors. **(A)** Detection of LMO7 expression in human primary and metastatic PC tumors. Immunohistochemical staining was used to detect LMO7 in human normal pancreas, primary PDAC, and metastatic PDAC in liver and lymph node. Red arrows point to ductal cells in normal pancreas and PDAC tumors. Weak staining of LMO7 in normal pancreas and strong staining in PDAC tumors were shown. Yellow arrows point to remarkable desmoplasia in primary and metastatic PDAC tumors. **(B)** Detection of LMO7 expression in human PNETs. Immunohistochemical staining was used to detect LMO7 in normal human pancreas, PNETs, peri-PNET tissue, and distant normal pancreas tissue. Red arrow points to islet in normal pancreas without positive staining of LMO7. On the contrary, a strong staining of LMO7 was detected in primary PNETs; a modest staining of LMO7 in peri-PNET tissue and distant normal pancreas tissue. PNET displayed a typical nested organoid pattern. **(C)** Western blot detected the expression of LMO7 in primary and metastatic human PDAC tumors. **(D)** Western blot detected the expression of LMO7 in PNETs and peri-PNET tissue. **(E)** LMO7 mRNA expression in 45 human PDAC tumors and peritumoral tissues. The paired PDAC tumors and adjacent tissues were harvested from 45 human patients. The significant increase in LMO7 mRNA expression was detected in the tumors compared to peritumoral tissues by qPCR. **(F)** qPCR detected LMO7 mRNA expression with the level that is higher in human Panc-1 cells than that in Mia-PaCa-2 cells. **(G)** qPCR detected the LMO7 expression with the level that is higher in mouse Panc02-H7 cells than that in Panc02 cells and UN-KPC-961 cells. **(H)** Schematic diagram of the establishment of orthotopic murine PC models in wild-type C57BL/6 mice. **(I)** The representative images show orthotopic murine PC models with or without liver metastasis induced with Panc02, Panc02-H7, or UN-KPC-961 cells. Yellow arrow points to orthotopic PC tumors without liver metastasis. Green arrow points to metastatic tumors in liver. **(J)** Western blot detects the strong expression of LMO7 protein in Panc02-H7 cells and its derived tumors in comparison to LMO7 expression in Panc02 and UN-KPC-961 cells as well as the derived tumors. *p < 0.05; **p < 0.01; ***p < 0.001.

To define the relationship between LMO7 expression and PC progression, the expression of LMO7 in primary and metastatic PC has been measured. In freshly harvested human pancreatic ductal adenocarcinoma (PDAC) and pancreatic neuroendocrine tumors (PNETs), we detected the enhanced LMO7 production in human PDACs and PNETs with IHC (Figures 1A,B) and Western blot (Figures 1C,D). Similar LMO7 expression patterns were observed in other 2 normal pancreas, 11 primary PDAC tumors, 2 metastatic PDAC in liver, and 1 metastatic PDAC in lung (Supplementary Figures 1A,B). Furthermore, qPCR detected the significantly increased expression of LMO7 mRNA in tumors from 45 human PC patients compared to that in relevant adjacent tissues (peritumoral tissues) (Figure 1E). Further analysis revealed that the increased LMO7 expression was mainly detected in tumors from the patients at stages I and II, but not stage III (Supplementary Figure 1C). Different levels of LMO7 mRNA expression were also detected in various types of human and mouse PC cells with expression levels higher in human Panc-1 cells (Figure 1F) and mouse Panc02-H7 cells (Figure 1G) than human Mia-Paca-2 cells and mouse Panc02 and UN-KPC-961 cells. Among them, Panc-1 and Panc02-H7 cells are more aggressive and able to induce tumor metastasis. By injecting cells into the pancreas of wild-type C57BL/6 mice (Figure 1H), we developed orthotopic PC-bearing mice with Panc02-H7, Panc02, or UN-KPC-961 cells. The formed PC tumors (yellow arrow) were detected in all recipient mice 17 days post cell injection (*n* = 5 for each group, Figure 1I), but tumor liver metastasis (green arrow) was only observed in the tumor-bearing mice developed with Panc02-H7 cells (middle panel in Figure 1I). Western blot detected the expression of LMO7 protein with a level that is higher in tumors developed with panc02-H7 cells than Panc02 cells or UN-KPC-961 cells (Figure 1J). Together, these results indicate that LMO7 mRNA and protein expression is consistently increased in human and mouse primary and metastatic tumors, suggesting their positive correlation with PC progression.

### Generation of Stable PC Cell Lines With LMO7 Knockdown and Knockout

**FIGURE 2:**
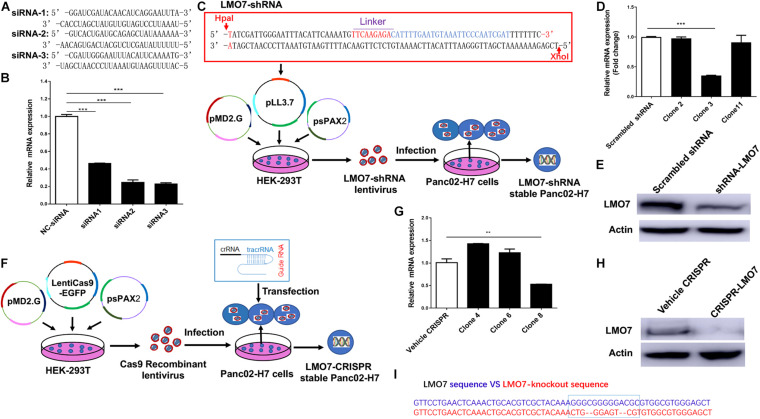
Knockdown or knockout of LMO7 in Panc02-H7 cells with siRNA, shRNA, and CRISPR-Cas9 technologies and establishment of relevant stable cells. **(A)** The sequences of three designed siRNAs for mouse LMO7. **(B)** qPCR detected the effective knockdown of LMO7 in Panc02-H7 cells with three designed siRNAs. **(C)** Schematic diagram of the establishment of stable LMO7-knockdown cells with the shRNA approach. **(D)** qPCR detected the reduced LMO7 mRNA expression in stable LMO7-knockdown cells developed with shRNA. **(E)** Western blot detected the reduced LMO7 protein expression in stable LMO7-knockdown cells developed with shRNA. **(F)** Schematic diagram of the establishment of stable LMO7-knockout cells with the CRISPR-Cas9 approach. **(G)** qPCR detected the reduced LMO7 mRNA expression in the CRISPR-induced stable LMO7-knockout cells. **(H)** Western blot showed an undetectable LMO7 protein expression in LMO7-CRISPR Panc02-H7 cells. **(I)** Sanger DNA sequencing shows the depleted nucleotides in the LMO7 gene in the stable knockout cells. **p < 0.01; ***p < 0.001.

To study the role of LMO7 in PC tumor progression, genetic methods with siRNA, shRNA, and CRISPR-Cas9 technologies were used to establish PC cells with LMO7 knockdown and knockout with our established method (Liu et al., 2019). In this regard, three independent genome-wide siRNAs were designed and transfected to Panc02-H7 cells (Figure 2A). A significant decrease of LMO7 mRNAs was detected in all three siRNA-transfected cells compared to the cells receiving control scrambled siRNAs (Figure 2B). Among three siRNAs, siRNA 2 and 3 showed a similar effect in strongly inducing LMO7 knockdown. Therefore, siRNA 3 was selected to use in the following experiments. In reference to siRNA 3, we designed LMO7-shRNA and inserted it into lentivirus vector pLL3.7. Co-transfection of this recombinant plasmid with helper vectors psPAX2 and pMD2G to HEK293T cells was used to package lentivirus particles with LMO7-shRNA. The resultant recombinant lentiviruses were infected into Panc02-H7 cells for establishing stable cells expressing LMO7-shRNA. A limited dilution method was used to generate single-cell clones (Figure 2C). qPCR and Western blot detected a significantly reduced expression of LMO7 at mRNA (Figure 2D) and protein (Figure 2E) levels in cell clone 3, which was named as LMO7-shRNA-Panc02-H7 cells. Using CRISPR-Cas9 technology, stable Panc02-H7 cells with LMO7 knockout were also generated with our successful protocol (Liu et al., 2019). We designed crRNA which was annealed with tracrRNA to get a guide RNA duplex which was transfected to the stable Cas9-Panc02-H7 cells in order to develop LMO7-knockout cells with CRISPR-Cas9 (Liu et al., 2019; Figure 2F). Limited dilution was used to isolate single-cell clones. qPCR and Western blot detected the significantly reduced expression of LMO7 mRNA (Figure 2G) and protein (Figure 2H) in cell clone 8, which was named as LMO7-CRISPR-Panc02-H7. Sanger DNA sequencing demonstrated nucleotide depletion in the LMO7 gene (Figure 2I). These results indicate that stable Panc02-H7 cells with LMO7 knockdown and knockout have been successfully established.

### Intrinsic LMO7 Defect Causes Suppression of Panc02-H7 Cell Proliferation, Colony Formation, and Motility *in vitro*

**FIGURE 3:**
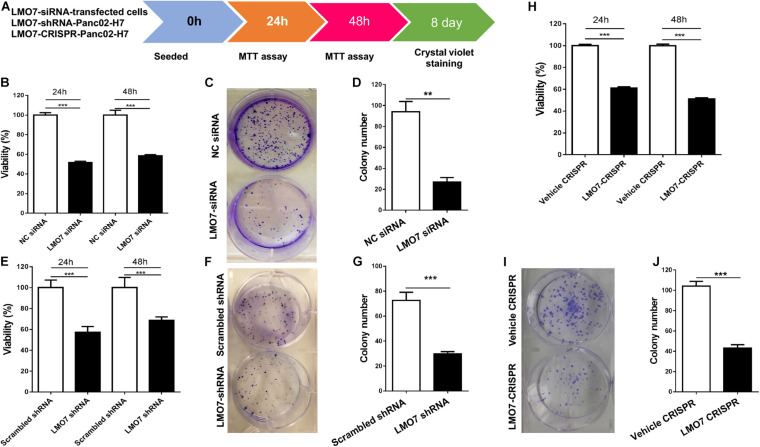
LMO7 defect-caused suppression of cell proliferation and colony formation in Panc02-H7 cells. **(A)** Schematic diagram for assay of LMO7-mediated cell proliferation and colony formation. **(B)** MTT detected the suppression of Panc02-H7 cell proliferation post knockdown of LMO7 with siRNAs. **(C)** Crystal violet staining detected the reduced cell colonies in Panc02-H7 cells post knockdown of LMO7 with siRNAs. **(E–G)** The reduced cell proliferation and colony formation in stable LMO7-shRNA-Panc02-H7 were detected with the same methods described in **(B)** to **(D)**. **(H–J)** The reduced cell proliferation and colony formation in stable LMO7-CRISPR-Panc02-H7 cells were detected with the same methods described in **(B)** to **(D)**. *n* = 3, error bars represent mean ± SD. **p < 0.01; ***p < 0.001.

**FIGURE 4:**
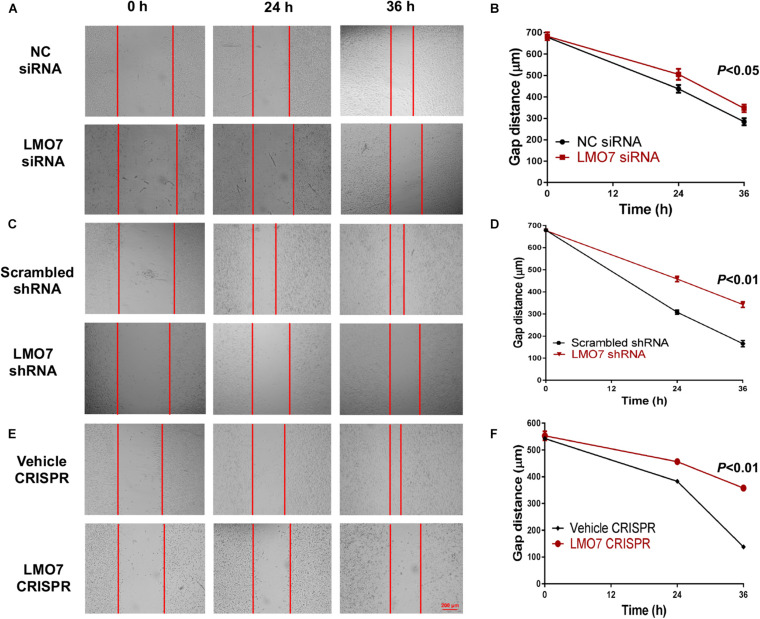
Silencing LMO7 suppresses Panc02-H7 cell motility. LMO7-siRNA-transfected Panc02-H7 cells, stable LMO7-shRNA-Panc02-H7 cells, or stable LMO7-CRISPR-Panc02-H7 cells were seeded into the 24-well plate with an insert at a dose of 2 × 10^5^ cells/well. On the second day, the inserts were removed and the cell-free gap was measured in the indicated times. Representative images showed the reduced widths of cell-free gaps in siRNA-knockdown Panc02-H7 cells **(A,B)**, stable LMO7-shRNA-Panc02-H7 cells **(C,D)**, or LMO7-CRISPR-Panc02-H7 cells **(E,F)** in comparison to that in the relevant control cells. *n* = 3, error bars represent mean ± SD.

Using stable Panc02-H7 cells with LMO7 knockdown and knockout, we investigated the role of intrinsic LMO7 defect in PC cell growth and tumorigenesis *in vitro* (Figure 3A). MTT examination revealed that siRNA-induced LMO7 knockdown significantly reduced Panc02-H7 cell proliferation (Figure 3B) and colony formation (Figures 3C,D) compared to control cells transfected with scramble siRNA. These effects were verified in stable LMO7-shRNA-Panc02-H7 cells with LMO7 knockdown (Figures 3E–G) and stable LMO7-CRISPR-Panc02-H7 cells with LMO7 knockout (Figures 3H–J). These results indicate that either transient or stable LMO7 knockdown or knockout significantly damages PC cell growth and tumorigenesis, suggesting its critical role in PC development. Next, we investigated if LMO7 defect also influences PC cell motility. For this purpose, the LMO7-siRNA-transfected Panc02-H7 cells, stable LMO7-shRNA-Panc02-H7 cells, and LMO7-CRISPR-Panc02-H7 cells were respectively seeded in 24-well plates with an inserter. On the second day, the inserters were removed. The cell-free gaps were measured under microcopy over the indicated times. As shown in Figures 4A,B, siRNA-mediated LMO7 knockdown significantly inhibited cell motility. The more obvious suppression of cell motility was observed in both stable LMO7-shRNA-Panc02-H7 cells (Figures 4C–D) and stable LMO7-CRISPR-Panc02-H7 cells (Figures 4E–F). LMO7 defect-mediated suppression on cell proliferation, colony formation, and cell motility were also detected in UN-KPC-961 cells post knockdown of LMO7 with shRNAs (Supplementary Figure 2). Together, the results indicate that loss of LMO7 function inhibits not only PC growth but also cell motility.

### Intrinsic LMO7 Defect Causes Slowed Tumor Growth *in vivo*

**FIGURE 5:**
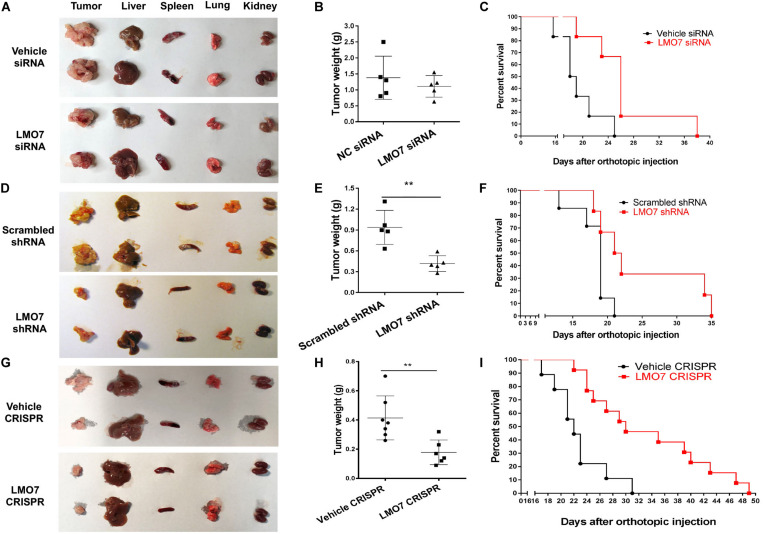
Silencing LMO7 in Panc02-H7 cells slows orthotopic tumor growth and extends the lifespan in recipient mice. LMO7-siRNA-transfected Panc02-H7 cells, stable LMO7-shRNA-Panc02-H7 cells, or stable LMO7-CRISPR-Panc02-H7 cells and corresponding control cells were injected into the head of the pancreas of mice at a dose of 2.5 × 10^5^ cells per mouse. Half of these mice were used to measure tumor size; the rest of them were used to measure the lifespan. 17 days post cell inoculation, some mice were euthanized for isolating tumors and different organs, and the tumors were weighed. Some mice were maintained to daily count survival over time, and the survival curves were made with Kaplan–Meier method. The representative images of tumors and different organs including spleen, liver, lung, and kidney in each mouse were shown **(A,D,G)**; accumulated tumor weights are shown in **(B,E,H)**, and viable mice in each group are shown in **(C,F,I)**. Error bars represent mean ± SD. **p < 0.01.

Next, we investigated whether intrinsic LMO7 defect in Panc02-H7 cells impacts tumor growth *in vivo*. LMO7-siRNA-transfected Panc02-H7 cells, LMO7-shRNA-Panc02-H7 cells, LMO7-CRISPR-Panc02-H7 cells, and corresponding control cells at a dose of 2.5 × 10^5^ cells per mouse were injected to the pancreas of wild-type mice at eight weeks of age. Seventeen days post cell injection, all mice were euthanized. No significant difference for body weight was detected in each mouse among groups. However, the mean volume of the tumors developed with Panc02-H7 cells with siRNA-induced LMO7 knockdown was smaller than the tumors developed with the control cells transfected with scrambled siRNAs, but this difference was not statistically significant (*n* = 5 for each group, *p* > 0.05, Figures 5A,B). In contrast, the tumors developed with stable LMO7-shRNA-Panc02-H7 or LMO7-CRISPR-Panc02-H7 cells were significantly smaller than those induced with the relevant control cells (*n* = 5 for each group, *p* < 0.01, Figures 5D–E, and *n* = 6 for LMO7-CRISPR cells, *n* = 7 for relative control, *p* < 0.01, Figures 5G–H). Also, no liver metastatic tumor was detected in the mice receiving inoculation of the cells with LMO7 defect (data not shown). The reduced LMO7 protein expression was observed in the tumors developed with LMO7-siRNA-transfected Panc02-H7 cells (Supplementary Figure 3A), stable LMO7-shRNA-Panc02-H7 cells (Supplementary Figure 3B), and stable LMO7-CRISPR-Panc02-H7 cells (Supplementary Figure 3C).

In a separate study, we examined whether intrinsic LMO7 knockdown or knockout impacts the lifespan of the recipient mice. After inoculation of the distinct cells with or without LMO7 knockdown and knockout, the development of ascites and impairment of gait and breathing were daily observed which is used to define the humane endpoint of the resultant tumor-bearing mice. Kaplan–Meier curves showed that the median life span of the mice receiving LMO7-siRNA-transfected cells is approximately 1.4-fold longer than that of the mice receiving control cells transfected with scrambled siRNAs (*n* = 6 for each group, Figure 5C). Similarly, the extended life span was also observed in the mice receiving stable LMO7-shRNA-Panc02-H7 cells compared to the mice (*n* = 6 for experimental group, *n* = 7 for the control group, Figure 5F). Correspondingly, a median life span of 30 days was observed in mice receiving stable LMO7-CRISPR-Panc02-H7 cells, which is much longer than 22 days in mice receiving relevant control cells. The log-rank test of significance across the groups has a *p* value < 0.001 (*n* = 13 for the experimental mice receiving LMO7-CRISPR-Panc02-H7 cells, *n* = 9 for the control mice receiving the relevant control cells, Figure 5I). These results suggest that intrinsic LMO7 defect not only slows tumor growth but also suppresses tumor metastasis, resulting in the significantly extended lifetime.

### LMO7 Knockout Causing Cell-cycle Arrest at the G1/S Phase and PC Cell Apoptosis

**FIGURE 6:**
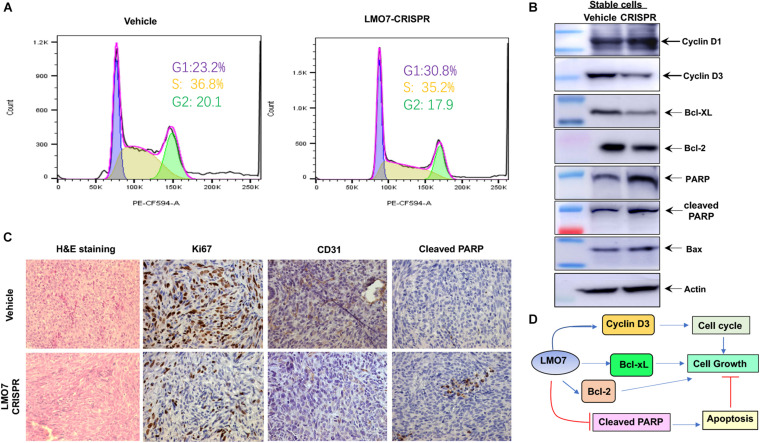
LMO7 defect causes cell-cycle arrest and cell apoptosis. **(A)** Cell-cycle arrest. Flow cytometric assay with PI detected the different frequencies of cells at phases G1, S, and G2 presented in stable LMO7-CRISPR-Panc02-H7 cells and the control cells. **(B)** Expression of canonical molecules important for cell cycle and apoptosis. Western blotting detected the decreased expression of cyclin D3, Bcl-XL, and Bcl-2 and increased expression of cyclin D1, PARP, cleaved PARP, and Bax in stable LMO7-CRISPR-Panc02-H7 cells in comparison to their control cells. **(C)** Expression of growth and apoptotic proteins in the tumors. The tumors were harvested and then fixed with formalin to prepare slides. H&E staining showed the tumor structure (left panel); IHC detected the reduced expression of Ki67 and CD31 (middle two panel) and increased expression of cleaved PARP (right panel). **(D)** A schematic diagram of LMO7-mediated signaling transduction pathways in PC.

To elucidate the molecular pathways underlying LMO7-mediated effects on PC tumorigenesis, we investigated how LMO7 knockout impacts Panc02-H7 cell cycle and apoptosis. As shown in Figure 6A, LMO7-CRISPR-Panc02-H7 cells with LMO7 knockout caused a cell-cycle arrest at the G1/S phase which was demonstrated by flow cytometric analysis by staining DNAs with propidium iodide (PI), suggesting regulated effect of LMO7 on cell cycle. Furthermore, we detected the impact of LMO7 knockout on expression of the well-characterized signaling molecules relevant with the cell cycle. Western blotting showed that LMO7 knockout markedly reduced the expression of cyclin D3, but not cyclin D1, in the LMO7-CRISPR-Panc02-H7 cells in comparison to the control cells (shown in Figure 6B), implying that LMO7 knockout induces cell-cycle arrest possibly by suppressing cyclin D3. Cyclin D3 is a member of the cyclin D family, which regulates the initial G1 to S transition (Kato, 1999). Next, we investigated if LMO7 defect-mediated cell-cycle arrest is linked to cell apoptosis (Pucci et al., 2000). For this purpose, we detected a panel of apoptotic signaling molecules in LMO7-CRISPR-Panc02-H7 cells. Western blot detected markedly increased the protein expression of PARP, cleaved PARP, and Bax and decreased the expression of Bcl2 and Bcl-xL in the LMO7-CRISPR-Panc02-H7 cells compared to control cells (Figure 6B). IHC detected the decreased expression of Ki67 and CD31 and increased the expression of cleaved PARP in the tumors developed with stable LMO7-CRISPR-Panc02-H7 cells compared to the tumors induced with control cells (Figure 6C). These results suggested that intrinsic LMO7 knockout suppresses PC tumorigenesis through coupling of the cell-cycle arrest and programmed cell death (Figure 6D).

## Discussion

Our *in vitro* and *in vivo* studies have demonstrated that LMO7 as an under-investigated factor significantly promotes PC tumorigenesis through influencing cell cycle and programmed cell death. Genetic knockdown or knockout of LMO7 not only significantly suppresses PC cell proliferation, colony formation, and motility *in vitro* but also obviously slows tumor growth and metastasis *in vivo*. This novel finding suggests that LMO7 represents a druggable molecule, which could be harnessed to develop a novel molecularly targeted therapy for PC.

To our knowledge, this is the first study demonstrating that LMO7 functions as an independent tumor-promoting factor, highly expressed in PC. We detected the increased expression of LMO7 in different types of human and mouse PC tumors in comparison with peritumoral and normal pancreas tissues. This increase is reflected in both LMO7 mRNA and protein levels, which has been validated by different methods including IHC, Western blot, and qPCR (Figure 1 and Supplementary Figure 1). These data indicate that PC progression is significantly accompanied by the enhanced production of LMO7. This finding is consistent with the studies on other cancers, where investigators identified highly expressed LMO7 in a significant percentage of colon, breast, liver, lung, pancreas, stomach, and prostate tumor tissues but very few in normal tissues (Kang et al., 2000). Human Protein Atlas showed that all human PC tumors have medium or high LMO7 expression and high expression is unfavorable. LMO7 as a member of the PDZ-LIM protein family is characterized by their PDZ and LIM domains; the combination of two functional domains in one protein has wide-ranging and multi-compartmental cell functions during development and homeostasis (Krcmery et al., 2010). Mis-regulation can lead to cancer formation and progression. Using genetic methods with the siRNA, shRNA, or CRISPR-Cas9 approach to induce loss of LMO7 function, we investigated the effect of LMO7 on PC tumorigenesis. Our *in vitro* experiments demonstrated that intrinsic LMO7 defect significantly suppresses PC cell proliferation (Figure 3 and Supplementary Figure 2); this effect has been verified by various types of human and mouse PC cell lines. By intrapancreatic injection of stable cells with LMO7 knockdown or knockout into wild-type C57BL/6 mice, we demonstrated that intrinsic LMO7 suppression slowed orthotopic PC tumor growth and metastasis (Figure 5). These *in vitro* and *in vivo* results imply that LMO7 functions as an intrinsic tumor-promoting factor in PC which provides a novel therapeutic target for PC treatment. Different from our findings in PC, Miyoshi et al. demonstrated that LMO7-deficient mice developed irregular and protruding epithelial lesion in the terminal and respiratory bronchioles at younger ages and tended to develop naturally occurring lung cancer at older ages. These results suggest that intrinsic and extrinsic LMO7 might exert different roles in tumorigenesis in a cancer type-dependent way (Tanaka-Okamoto et al., 2009). To address this question, we will use LMO7-knockout mice to investigate whether extrinsic LMO7 similarly affects PC and elucidate the underlying mechanisms.

Our studies suggest that LMO7 as a critical factor is involved in PC metastasis. The Panc02-H7 cell line is derived from non-metastatic Panc02 cells by continuously passaging the cells to wild-type mice as selection pressure (Wang et al., 2003). Using qPCR and Western blot, we detected the higher expression of LMO7 at either mRNA or protein level in Panc02-H7 cells compared to Panc02 cells (Figure 1G). Intrapancreatic inoculation of both Panc02-H7 cells and Panc02 cells into wild-type mice induces orthotopic PC tumors, but tumor liver metastasis was only detected in the mice receiving Panco2-H7 cells (Figures 1H,I). These results reveal the positive correlation between LMO7 expression and PC tumor metastasis. Next, we used different Panc02-H7 cells with LMO7 knockdown or knockout to investigate whether LMO7 is critical to mediate PC metastasis. As described in Figure 4, LMO7 defect induced with siRNA, shRNA, and CRISPR-Cas9 approaches suppresses Panc02-H7 cell motility *in vitro*. Consistent with our finding, one group previously reported that the expression of LMO7 was detected in metastatic breast cancer cell line MDA-MB-231, but not in non-metastatic MCF7 cells (Hu et al., 2011). Using transwell migration assays, they demonstrated that LMO7 knockdown markedly reduced the migration of MDA-MB-231 breast cancer cells by specifically regulating MRTF in a cell-specific manner (Hu et al., 2011). Mechanistic studies revealed that LMO7 enhances cell migration by activating Rho and MRTF. These data suggest that LMO7 as an independent factor drives tumor metastasis in different cancers including PC (Ooshio et al., 2004; Pipes et al., 2006). The mechanisms underlying LMO7-mediated PC metastasis remain to be understood.

In this study, we have successfully developed *in vitro* and *in vivo* cancer models to study the role and mechanisms of LMO7 in PC. Using siRNA, shRNA, and CRISPR-Cas9 technologies, we generated stable PC cells with LMO7 knockdown or knockout (Figure 2). These cells have been used to induce orthotopic PC tumors in wild-type C57BL/6 mice (Figure 5). These cells and animal models complementarily provide us ideal platforms to mechanistically study LMO7’s important role in PC. We have demonstrated that LMO7 knockout causes not only cell-cycle arrest (Figure 6A) but also cell apoptosis (Figure 6B), suggesting that LMO7 has dual but related functions by linking cell-cycle progression and programmed cell death (Figure 6D). Single protein-acting dual-signal models have been found in many other molecules such as tumor-suppressor genes p53 and Rb, the dominant oncogenes c-Myc, and several cyclin-dependent kinases (Cdks) and their regulators (Pucci et al., 2000). For example, *p53* is recognized as one of widely studied tumor suppressors. As a nuclear DNA-binding phosphoprotein, *p53* influences cell proliferation by acting predominately in the G1 phase of the cell-cycle progression. In addition, *p53* also plays an important role in triggering apoptosis in certain cell types. It has been reported that LMO7 as an emerin-binding protein regulates its transcription (Holaska et al., 2006). One group has demonstrated that emerin is a nuclear protein gene important for maintaining cell-cycle timing (Fairley et al., 2002). Together, LMO7 induces cell-cycle arrest likely through regulation of cyclin D3 and emerin. We will test this hypothesis in the future studies.

In this study, we have established stable PC cells with LMO7 knockdown and knockout. Using these cells as a platform, we have demonstrated that intrinsic LMO7 defect causes significant suppression of tumor growth and metastasis by inducing PC apoptosis and cell-cycle arrest at the G1/S phase. These results suggest that LMO7 functions as an oncogene in PC. Targeting this under-investigated molecule offers us an opportunity to develop a new molecularly targeted therapy for PC.

## Materials and Methods

### Antibodies, Reagents, and Plasmids

Antibodies against PARP, cleaved PARP, Bcl-2, Bcl-XL, Bax, β-actin, Cyclin D1, Cyclin D3, Ki67, and CD31 were bought from Cell Signaling (Danvers, MA, United States). Mouse LMO7 antibody was purchased from Santa Cruz (California, CA, United States). All immunohistochemistry (IHC) reagents including ImmPRESS^TM^ HRP anti-rabbit IgG (Peroxidase) (Cat#MP-7401), ImmPACT DAB peroxidase (HRP) substrate (Cat#SK-4105), and hematoxylin (Cat#H-3404) were purchased from Vector Laboratories (Burlingame, CA, United States). Plasmids pLL3.7 and LentiCas9-EGFP (Cat#63592) were bought from Addgene (Cambridge, MA, United States).

### Cell Lines, Medium, and Culture

Murine Panc02-H7 cells were a generous gift from Dr. Keping Xie in MD Anderson Cancer Center, which is an invasive cell line derived from Panc02 (Wang et al., 2003). The UN-KPC-961 cell line was purchased from ATCC (Manassas, VA); this cell was generated from pancreatic tumors in 17-week-old Pdx1-Cre (KPC) mice with mutation of Kras (G12D) and Trp53 (R172H). Human Panc-1 and MIA PaCa-2 cells were purchased from ATCC (Manassas, VA, United States). The cell lines were maintained in Dulbecco’s Modified Eagle Medium (DMEM; Cellgro, Manassas, VA, United States) supplemented with 100 U/mL penicillin, 100 μg/mL streptomycin, 2 mmol/L L-glutamine, 10 mmol/L HEPES, and 10% fetal bovine serum (FBS) at 37°C in a 5% CO_2_ humidified atmosphere (Qi et al., 2016).

### Mice

Six-week-old male C57BL/6 mice were purchased from Jackson Laboratory (Bar Harbor, ME). Mice were housed under standard conditions with a 12-h light/12-h dark cycle with room temperature 21 ± 2°C in the first floor of the Medical Sciences Building at the University of Missouri-Columbia. This space is an AAALAC- and USDA-accredited state-of-the-art facility and administered by the Animal Care Unit at the University of Missouri. Mice were maintained in individually ventilated cages (IVC) with free access to water and standard mouse diet (PicoLab Rodent Diet 20, 21% protein, 11.3% fat). All experiments with mice were performed under a protocol approved by the Institutional Animal Care and Use Committee (IACUC) at University of Missouri. All mice received humane care according to the criteria outlined in the “Guide for the Care and Use of Laboratory Animals.” Animals are free of clinical signs of disease before surgery. After surgery, mice were immediately kept on a warm pad in a dry area and vital signs will be monitored during recovery. After recovery from anesthesia, the mice were returned to routine housing. Animals are monitored daily, and mice that became ill during these experiments will be euthanized.

### Human PC Tumor Samples

The protocol (IRB2010166) for freshly harvesting human PC tumors and adjacent tissues in Ellis Fischel Cancer Center was approved by the Institutional Review Board (IRB) at the University of Missouri School of Medicine. All subjects gave their informed consent for inclusion before they participated in the study. Presence of tumor was confirmed by a pathologist with hematoxylin and eosin (H&E) staining. Other 45 human PC tumor tissues and corresponding adjacent tissues were collected at the Pancreas Center, the First Affiliated Hospital of Nanjing Medical University in Nanjing, China. The protocol (2017-SR-171) was approved by the Ethics Committee in this hospital. All of these patients underwent pancreatic resection and promised to provide their tumors with the informed consent. None of them received chemotherapy and/or radiotherapy before surgery. The tissues were frozen at −80°C within 10 min after tumor excision (He Y. et al., 2018; Hu et al., 2018).

### RNA Extraction and Quantitative PCR (qPCR)

Total RNAs were extracted using TRIzol reagent (Invitrogen, Carlsbad, CA) in terms of the manufacturer’s instruction. Reverse transcription of RNA to cDNA was conducted with High-Capacity cDNA Reverse Transcription Kits (Applied Biosystems, Foster City, CA, United States). qPCR was performed with QuantStudio 3 Detection System (ABI, Thermo Fisher) in a 20-μL reaction mixture containing SYBR Green I (Applied Biosystems, Foster City, CA, United States). The expression level of LMO7 was normalized to housekeeping gene of 18s rRNA and was further analyzed using the 2^–ΔΔCT^ method. The sequences of forward and reverse primers for human LMO7 are 5′-AATCAGCATAAACCAGACGCC-3′ and 5′-CTGGGCTACCTGCTTCAACT-3′; for 18S rRNA: 5′-AATCAGGGTTCGATTCCGGA-3′ and 5′-CCAAGATCCA ACTACGAGCT-3′.

### Orthotopic PC Murine Model, Tumor Weight, and Mouse Lifespan Analysis

PC cells grown to 90% confluence were harvested and suspended in 15% Matrigel in PBS. The suspended cells were seeded into the pancreas to make orthotopic PC models with our established protocol which was conducted in the biosafety cabinet of mouse vivarium (Liu et al., 2019). Briefly, male C57BL/6 mice (22.1 ± 1.8 g, aged 8 weeks) were anesthetized with VetEquip IMPAC by inhalation of 2% isoflurane (VetOne^®^ Fluriso^TM^, Boise, IL) mixed with oxygen at a flow of 1.5 L/min. Isoflurane caused faster induction of anesthesia and quick recovery as well as relative sparing effect on cardiovascular function. Adequate anesthesia was checked by the toe-pinch method. The anesthetized mice were placed on a temperature-controlled heating pad to induce vasodilatation in a ventilated restrainer immobilization for surgery. A 1.5-cm incision was made in the left flank to expose the pancreas. Mice were randomized into two groups for each experiment; each group contained 5–13 mice. 40-μl suspended cells or relevant control cells were injected into the head of the pancreas at a dose of 2.5 × 10^5^ cells/mouse. After cell injection, two layers of the flank incision were sutured and the skin was clipped. Intraperitoneal injection of 100 μl Carprofen at a dose of 5 mg/kg was applied for mouse pain alleviation after surgery. Pain and distress of mice were monitored daily. Development of ascites and impairment of gait and breathing were used to define a humane endpoint of the resultant tumor-bearing mice. At the endpoint, mice were euthanized by CO_2_ exposure with a flow of 3 L/min followed by cervical dislocation. A survival curve was constructed with the Kaplan–Meier method using GraphPad Prism software. Statistical significance was determined by single-factor analysis of variance and validated using the log-rank test. *p* values of <0.05 was considered significant.

### siRNA Transfection

Three predesigned siRNAs for mouse LMO7 were bought from Integrated DNA Technologies, Inc., (IDT, Coralville, IA). siRNA-mediated LMO7 knockdown in PC cells was conducted with our established method (Liu et al., 2019). Briefly, PC cells grown to 50% confluence in a 6-well plate received transfection of 5 pmol of siRNAs with RNAi-MAX Lipofectamine reagent (Invitrogen, Carlsbad, CA). Eight hours post transfection, the medium was changed to complete DMEM medium. The cells were cultured for another 36 h then harvested for the subsequent assay.

### Generating Stable LMO7 Knockdown Cells With shRNA

We used our reliable method to generate a stable LMO7 knockdown cell line (Liu et al., 2019). LMO7-shRNA or scrambled shRNA was designed, synthesized, annealed to form double-chain DNA fragments, then inserted into a vector plasmid pLL3.7 between *Hpa*I and *Xho*I sites. The recombinant plasmids with LMO7-shRNA or scramble shRNA were transformed into competent *Escherichia coli* cells and identified with PCR and DNA sequencing. The expanded recombinant plasmids pLL-shRNA-LMO7 or control pLL-shRNA-CTR, together with helping plasmids psPAX2 and pMD2G, were co-transfected into HEK 293T cells to package the lentiviruses. The supernatants containing the relevant lentivirus were harvested 48 h and 72 h post transfection and concentrated using the Lenti-X Concentrator (Clontech, Fremont, CA, United States). The recombinant lentiviruses were first transfected to the Panc02-H7 cells or UN-KPC-961 cells grown to 50% confluence. The medium was changed to the normal culture medium 12 h post infection. A limited dilution method was used to generate single-cell clones. Each clone was amplified to measure the mRNA expression level of LMO7 with qPCR. The resultant stable cell clone with a significant LMO7 suppression was named as LMO7-shRNA-Panc02-H7 cells or LMO7-shRNA-UN-KPC-961 cells.

### Generating Stable LMO7-Knockout Cells With the CRISPR-Cas9 Approach

We used our reliable method to generate stable LMO7 knockout cells (Liu et al., 2019). The tracrRNAs and crRNAs specific for LMO7 were designed by CRISPR DESIGN^1^ and synthesized by IDT (Coralville, IA). 1 μl crRNA (100 μM) and 1 μl tracrRNA (100 μM) were mixed with 18 μl nuclease-free water and heated to 95°C for 5 min, then cooled down to room temperature in order to make a guide RNA (gRNA) duplex. The gRNA duplex was transfected to the established Cas9-Panc02-H7 stable cells via the RNAi-MAX Lipofectamine reagent (Liu et al., 2019). Single-cell clones were generated with the limited dilution as in the above description. Each single clone was expanded, and genomic DNA for Sanger DNA sequencing was extracted. LMO7 knockout was verified with qPCR and Western blotting at the mRNA and protein level. The resultant cells with confirmed LMO7 knockout were named as LMO7-CRISPR-Panc02-H7 cells.

### Cell Proliferation Assay

Different PC cells with or without LMO7 knockdown and knockout were seeded in 96-well plates at a dose of 2 × 10^3^ per well. At the indicated time, cell proliferation was measured with Proliferation Assay Kit (Promega, Madison, WI, United States) according to the manufacturer’s instructions.

### Colony Formation Assay

Different PC cells with or without LMO7 knockdown and knockout were seeded in 6-well plates at a dose of 2 × 10^2^ per well. Seven to ten days later, the cells were rinsed with phosphate-buffered saline (PBS), then stained with 0.05% crystal violet for photography and colony counting.

### Wound Healing Assay

Different PC cells with or without LMO7 knockdown and knockout were seeded in 24-well plates with an insert at a dose of 2 × 10^5^ per well. The inserts were moved carefully on the second day, and cells were continued to culture. The cell-free gaps were measured many times with an optical microscope (Zeiss).

### Western Blotting Analysis

Cell lysate and tumor lysates were respectively prepared with lysis protein extraction reagent (Thermo Fisher Scientific, Inc.) and M-PERTM mammalian protein extraction reagent (Thermo Fisher Scientific, Inc.). After quantitating, an equal amount of protein was loaded to perform Western blotting as previously described (Liu et al., 2019).

### Cell-cycle Analysis

Cells with or without LMO7 knockout were harvested from the cultured dishes and suspended in PBS containing 50 μg/mL propidium iodide (PI) for flow cytometry. CellQuest software (BD Biosciences, San Jose, CA, United States) was used to determine cell-cycle distribution.

### Immunohistochemistry (IHC)

As described in our literature (Liu et al., 2019), tumor tissues harvested from tumor-bearing mice were sequentially fixed, dehydrated, and embedded to make 4-μm tissue sections. Tissue sections were used to conduct IHC by sequential de-paraffinization, incubation with primary antibody and secondary antibody, and final color development with DAB substrate. Three random fields in each slide were photographed under the microscope.

### Statistics Analysis

Paired data were analyzed using a 2-tailed paired Student’s *t*-test. A *p* value of <0.05 was considered significant.

## Data Availability Statement

The raw data supporting the conclusions of this article will be made available by the authors, without undue reservation.

## Ethics Statement

This study was performed in accordance with the Declaration of Helsinki. All experiments with mice were performed under a protocol approved by the Institutional Animal Care and Use Committee (IACUC) at University of Missouri. All mice received humane care according to the criteria outlined in the “Guide for the Care and Use of Laboratory Animals.” Informed consent was obtained from all patients, and this study was approved by the Ethics Committee of the hospital (2017-SR-171) at The First Affiliated Hospital of Nanjing Medical University, China.

## Author Contributions

XL, HY, and JZ performed study design, acquisition of data, analysis and interpretation of data, and statistical analysis. QW, XQ, and CB performed study design and data analysis. DA and JK performed study design and critical revision of the manuscript for important intellectual content. EK, KC, and YM performed critical revision of the manuscript for important intellectual content. PT performed pathologist to review the H&E and IHC slides and critical revision of the manuscript for important intellectual content. KJ performed study concept and design, analysis and interpretation of data, drafting of the manuscript, and critical revision of the manuscript for important intellectual content. GL performed study concept and design, analysis and interpretation of data, drafting of the manuscript, critical revision of the manuscript for important intellectual content, obtained funding, and study supervision. All authors contributed to the article and approved the submitted version.

## Conflict of Interest

The authors declare that the research was conducted in the absence of any commercial or financial relationships that could be construed as a potential conflict of interest.

## Footnotes

1 http://crispr.mit.edu/

## Supplementary Material

Supplementary Figure 1 Increased expression of LMO7 protein and mRNA in human PC tumors. **(A)** Tissue array of LMO7 immunohistochemical staining in extra 2 normal pancreas, 11 primary PDAC tumors, 2 metastatic PDAC in liver and 1 metastatic PDAC in lung. **(B)** Weak staining of LMO7 in normal pancreas and strong staining in PDAC tumors were shown. **(C)** LMO7 mRNA upregulation was detected in the human PC patients at early stages. Further analysis of Figure 1E results revealed the increased LMO7 expressions in tumors from the patients at the stage I and II, but not stage III.

Supplementary Figure 2 LMO7 defect causes suppression of UN-KPC-961 cell proliferation, colony formation and motility. **(A)** The reduced LMO7 protein expression in the shRNA-induced stable LMO7-knockdown UN-KPC-961 cells. **(B)** The reduced cell proliferation in stable LMO7-shRNA-UN-KPC-961 cells. **(C,D)** The reduced colony formation in stable LMO7-shRNA-UN-KPC-961 cells. **(E,F)** Silencing LMO7 suppresses UN-KPC-961 cells motility. Representative images **(E)** and average widths **(F)** of cell-free gaps were shown for LMO7-shRNA-UN-KPC-961 cells. **p* < 0.05; ***p* < 0.01; ****p* < 0.001.

Supplementary Figure 3 LMO7 protein expression in orthotopic tumors induced with LMO7 silenced Panc02-H7 cells. The reduced LMO7 protein expression was observed in the tumors developed with LMO7-siRNA-transfected Panc02-H7 cells **(A)**, stable LMO7-shRNA-Panc02-H7 cells **(B)**, and stable LMO7-CRISPR-Panc02-H7 cells **(C)**.
