# Supplementary material for: LMO7 as an Unrecognized Factor Promoting Pancreatic Cancer Progression and Metastasis
Source: Front Cell Dev Biol. 2021 Mar 8;9:647387. doi: 10.3389/fcell.2021.647387 (PMC7982467; doi:10.3389/fcell.2021.647387)
Supplement: Supplementary Figure 1 — Increased expression of LMO7 protein and mRNA in human PC tumors. (A) Tissue array of LMO7 immunohistochemical staining in extra 2 normal pancreas, 11 primary PDAC tumors, 2 metastatic PDAC in liver and 1 metastatic PDAC in lung. (B) Weak staining of LMO7 in normal pancreas and strong staining in PDAC tumors were shown. (C) LMO7 mRNA upregulation was detected in the human PC patients at early stages. Further analysis of Figure 1E results revealed the increased LMO7 expressions in tumors from the patients at the stage I and II, but not stage III. [file Table_1.docx]

***Supplementary Figures***


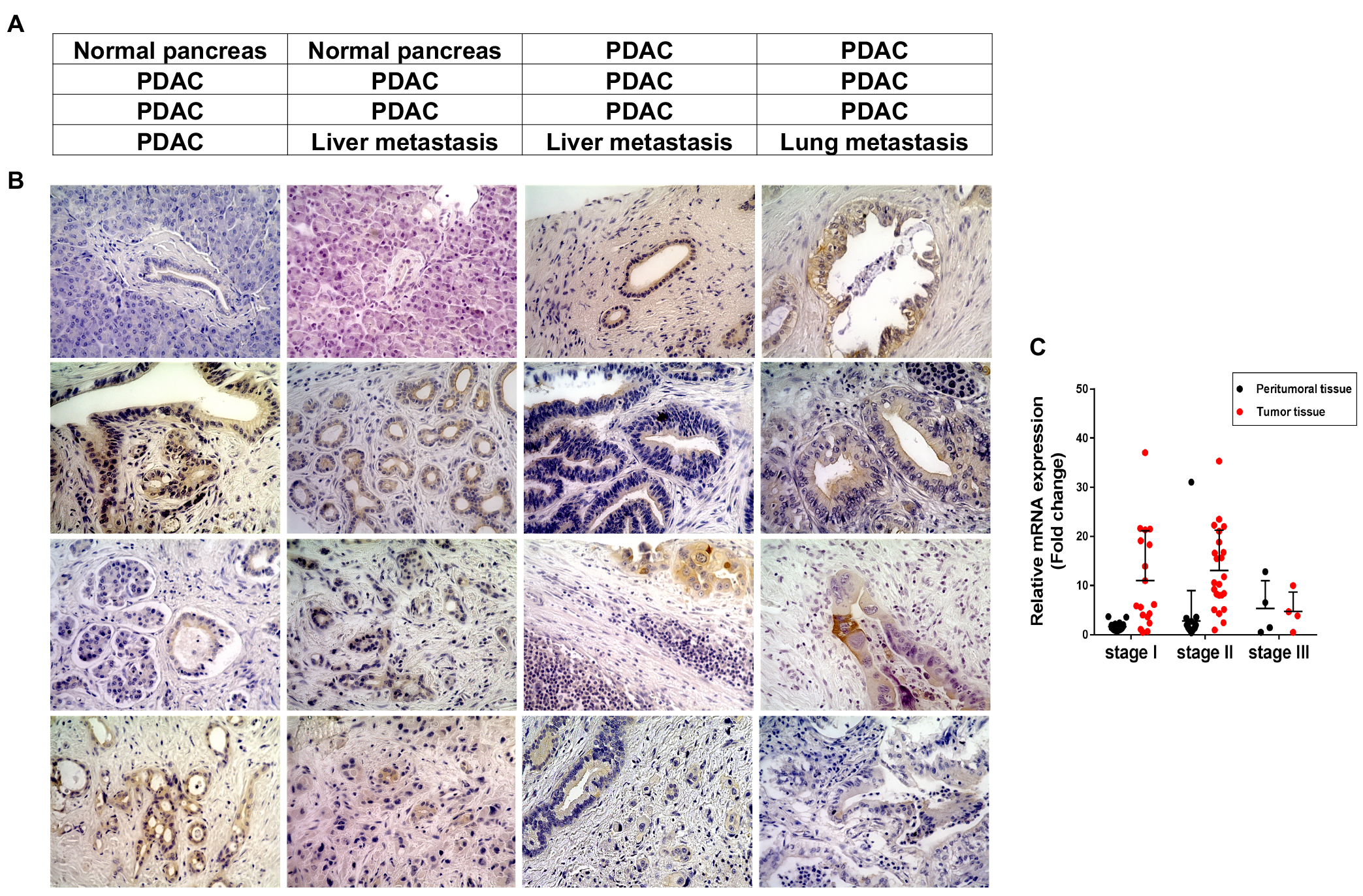


**Supplementary figure 1. Increased expression of LMO7 protein and mRNA in human PC tumors.** (A) Tissue array of LMO7 immunohistochemical staining in extra 2 normal pancreas, 11 primary PDAC tumors, 2 metastatic PDAC in liver and 1 metastatic PDAC in lung. (B) Weak staining of LMO7 in normal pancras and strong staining in PDAC tumors were shown. (C) LMO7 mRNA upregulation was detected in the human PC patients at early stages. Further analysis of figure 1E results revealed the increased LMO7 expressions in tumors from the patients at the stage I and II, but not stage III.


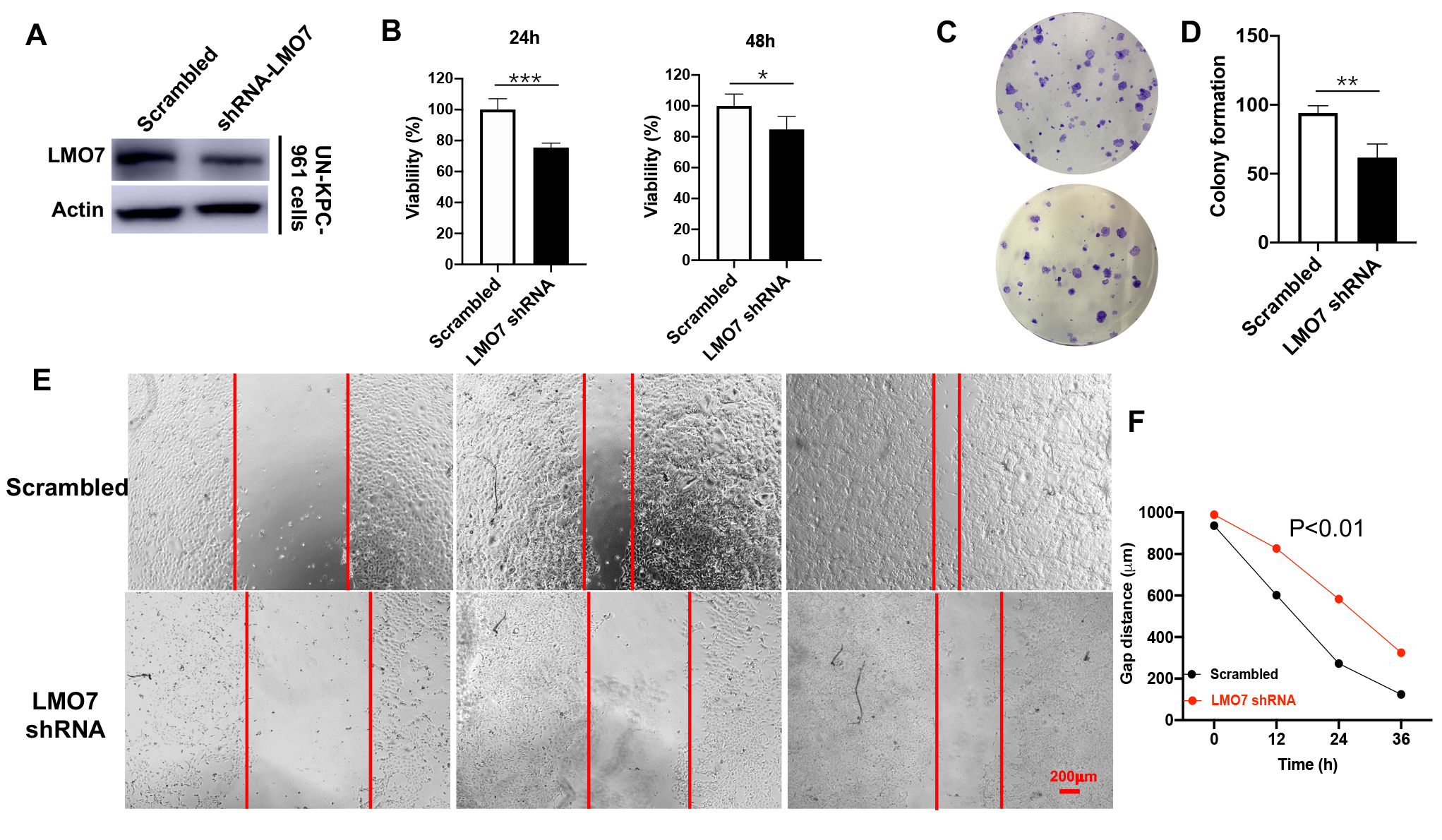


**Supplementary figure 2. LMO7 defect causes suppression of UN-KPC-961 cell proliferation, colony formation and motility.** (A) The reduced LMO7 protein expression in the shRNA-induced stable LMO7-knockdown UN-KPC-961 cells. (B) The reduced cell proliferation in stable LMO7-shRNA-UN-KPC-961 cells. (C and D) The reduced colony formation in stable LMO7-shRNA-UN-KPC-961 cells. (E and F) Silencing LMO7 suppresses UN-KPC-961 cells motility. Representative images (E) and average widths (F) of cell-free gaps were shown for LMO7-shRNA-UN-KPC-961 cells. ****p value < 0.0001, **p value < 0.01, and *p value < 0.05.


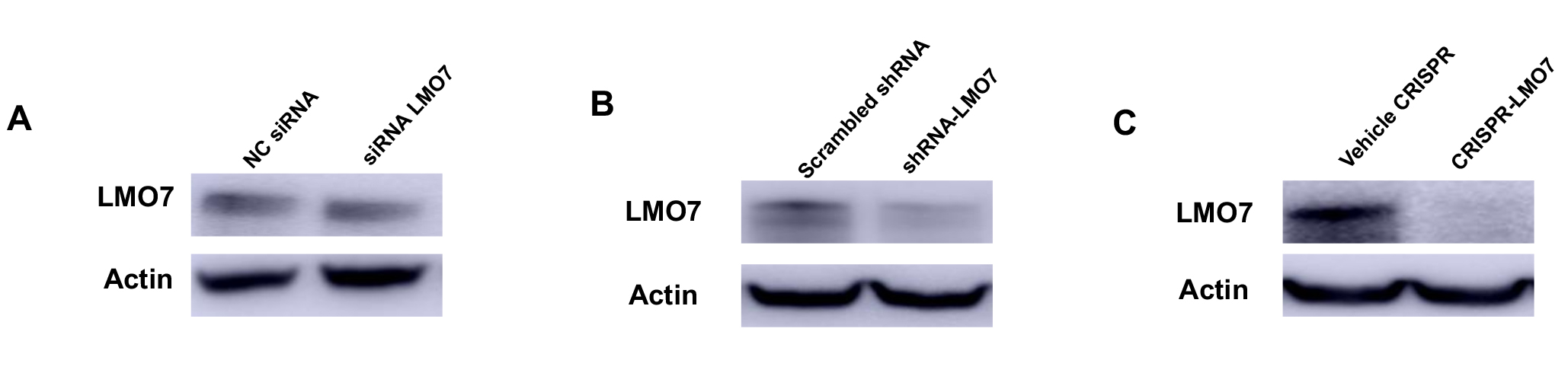


**Supplementary figure 3. LMO7 protein expression in orthotopic tumors induced with LMO7 silenced Panc02-H7 cells.** The reduced LMO7 protein expression was observed in the tumors developed with LMO7-siRNA-transfected Panc02-H7 cells (A), stable LMO7-shRNA-Panc02-H7 cells (B), and stable LMO7-CRISPR-Panc02-H7 cells (C).
